# Supplementary material for: Community Structures and Antifungal Activity of Root-Associated Endophytic Actinobacteria of Healthy and Diseased Soybean
Source: Microorganisms. 2019 Aug 7;7(8):243. doi: 10.3390/microorganisms7080243 (PMC6724048; doi:10.3390/microorganisms7080243)
Supplement: Supplementary file 1 [file microorganisms-07-00243-s001.pdf]

**Supporting Imformation**

# Isolation and Comparison of Root-associated Endophytic Actinobacteria from Healthy and Diseased Soybean and Identification of the Antifungal Compounds against *Sclerotinia sclerotiorum* (Lib.) de Bary

**Chongxi Liu[1,2], Xiaoxin Zhuang[1], Zhiyin Yu[1,2], Zhiyan Wang[2], Yongjiang Wang[2], Xiaowei Guo[1,2], Wensheng Xiang[1,3*], Shengxiong Huang[2*]**

[1]Key Laboratory of Agriculture Biological Functional Gene of Heilongjiang Provincial Education Committee, Northeast Agricultural University, Harbin 150030, China

[2]State Key Laboratory of Phytochemistry and Plant Resources in West China, Kunming Institute of Botany, Chinese Academy of Sciences, Kunming 650201, China

[3]State Key Laboratory for Biology of Plant Diseases and Insect Pests, Institute of Plant Protection, Chinese Academy of Agricultural Sciences, Beijing, China

*Correspondence: Wensheng Xiang and Shengxiong Huang

E-mail: xiangwensheng@neau.edu.cn, sxhuang@mail.kib.ac.cn.

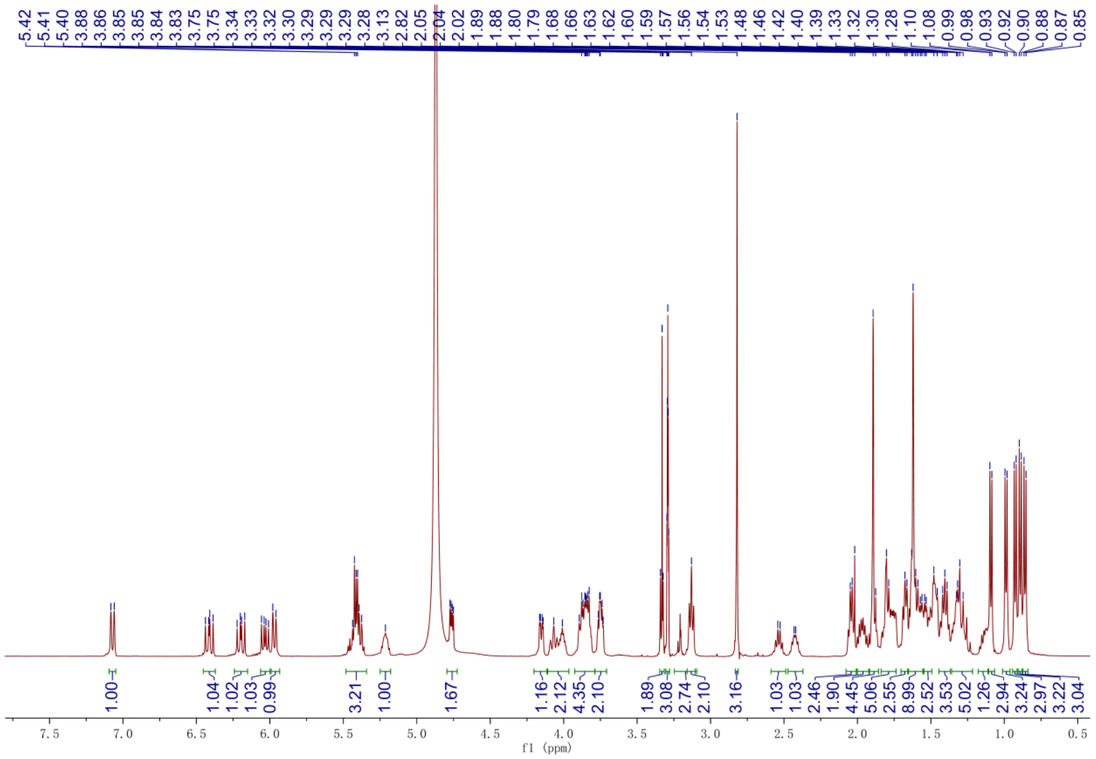


**Figure S1.** $^{1}$H NMR (600 MHz) spectrum of compound **1** in MeOH-$d_4$

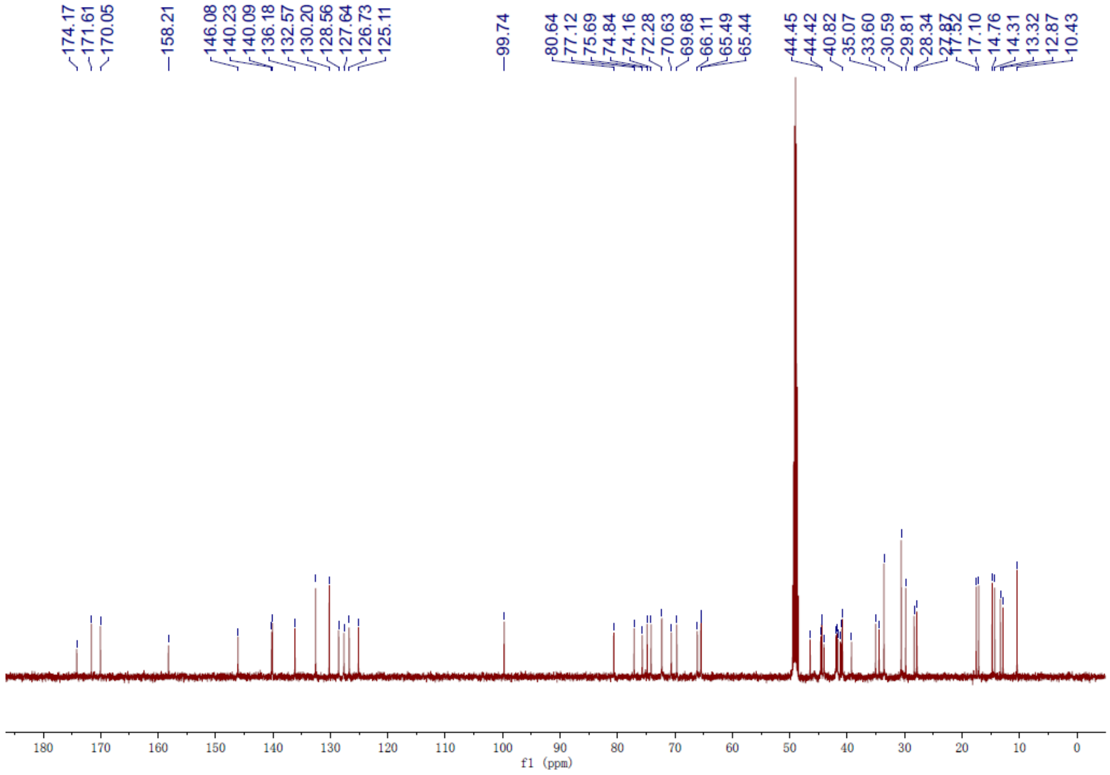


**Figure S1.** $^{13}$C NMR (600 MHz) spectrum of compound **1** in MeOH-$d_4$

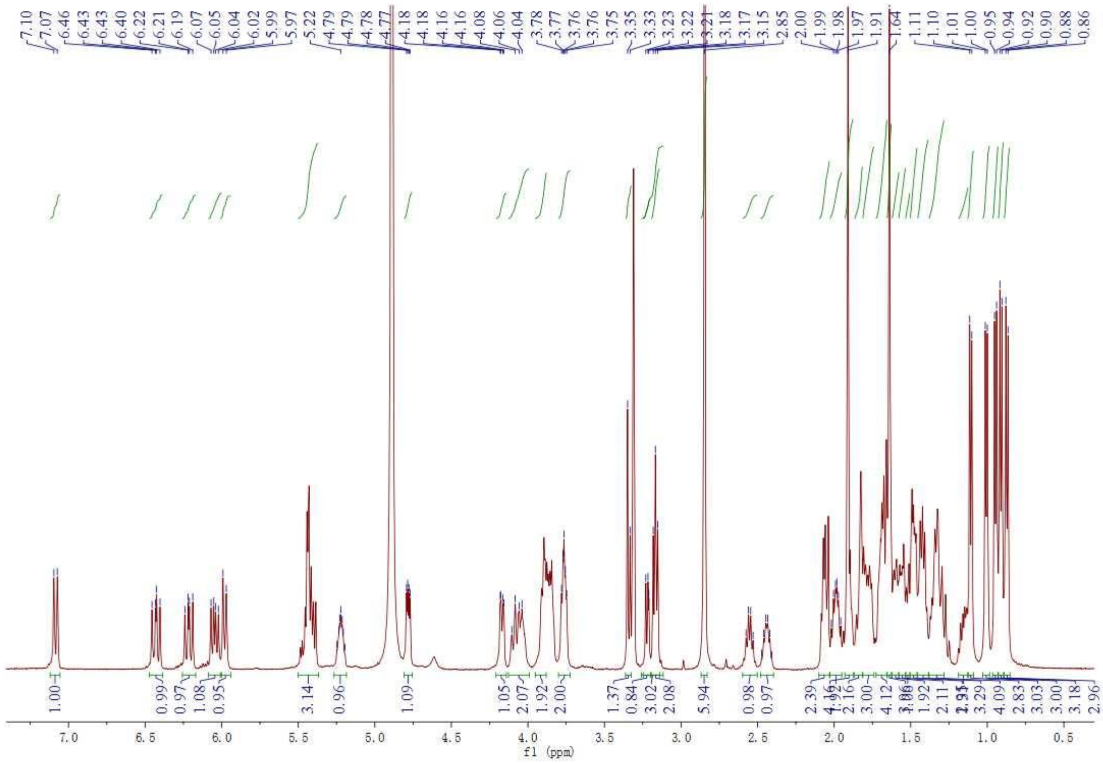

**Figure S1.** $^{1}$H NMR (600 MHz) spectrum of compound **2** in MeOH-$d_4$

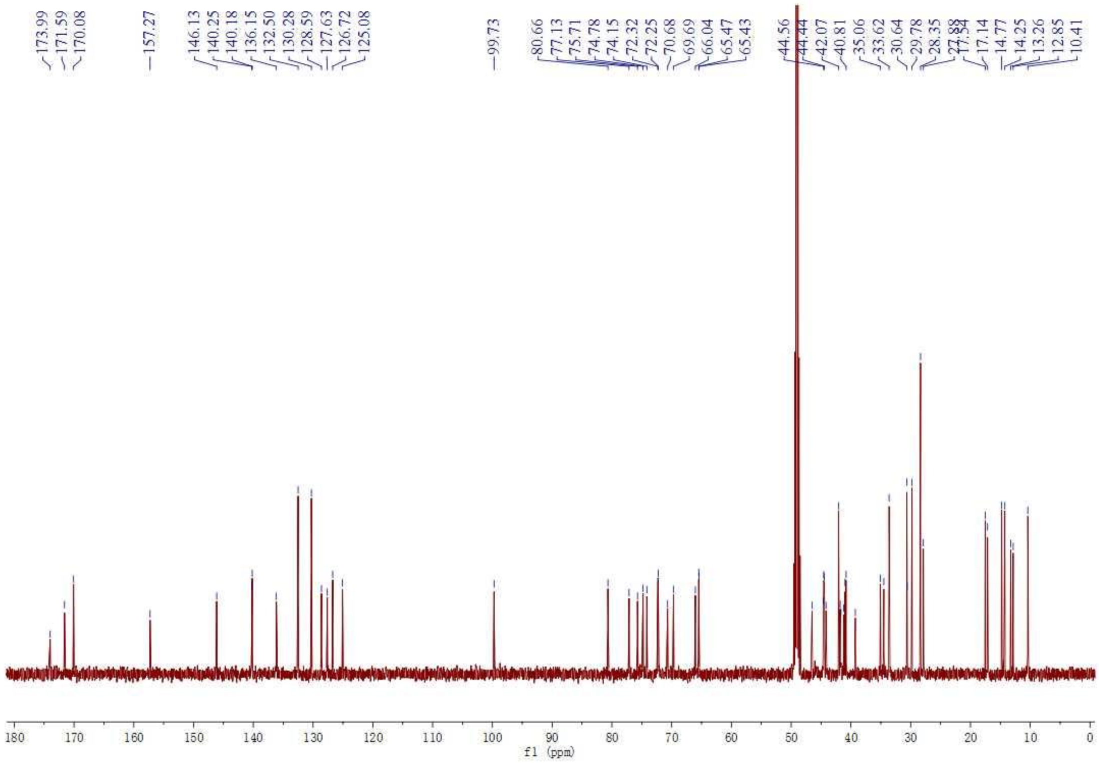

**Figure S1.** $^{13}$C NMR (600 MHz) spectrum of compound **2** in MeOH-$d_4$

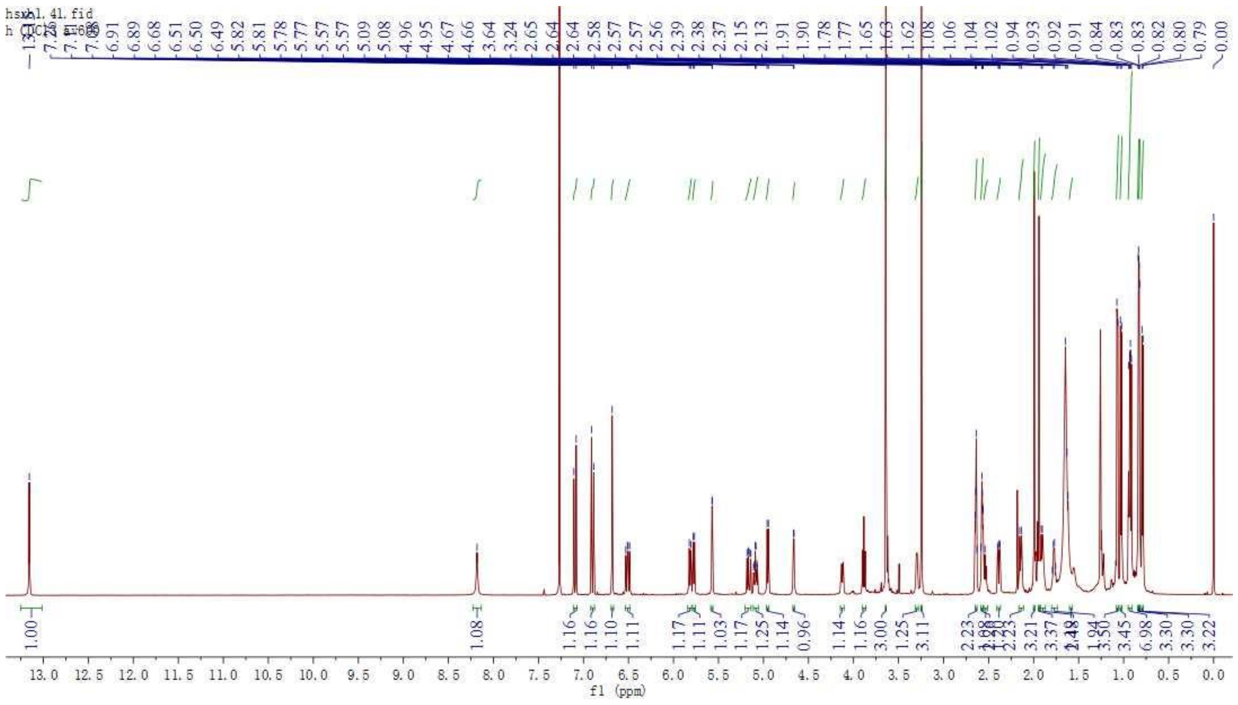


**Figure S1.** $^{1}$H NMR (600 MHz) spectrum of compound **3** in $CDCl_3$

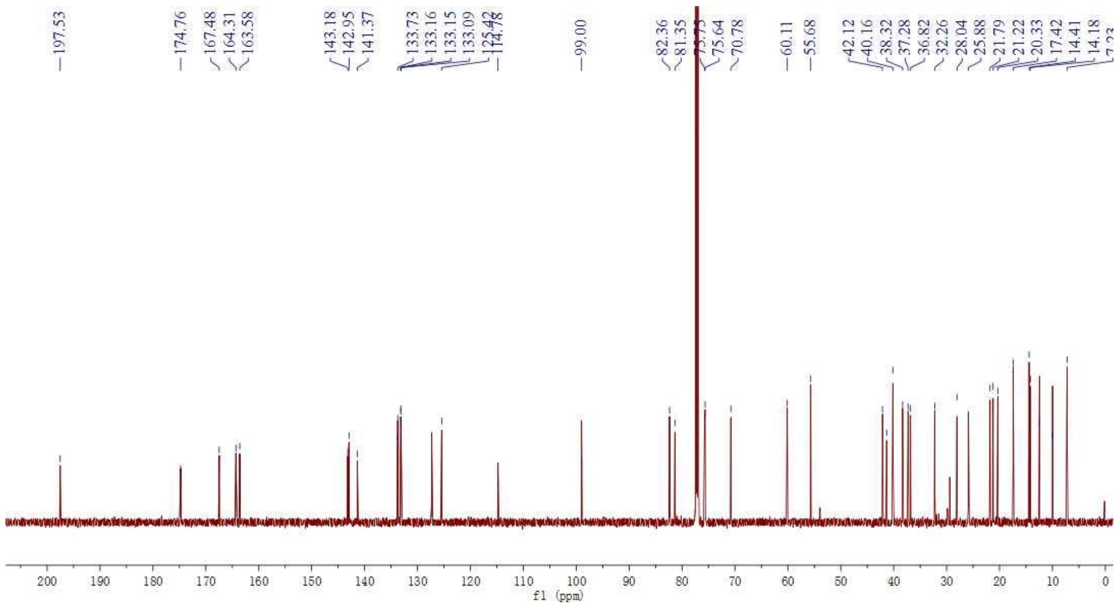


**Figure S1.** $^{13}$C NMR (600 MHz) spectrum of compound **3** in $CDCl_3$

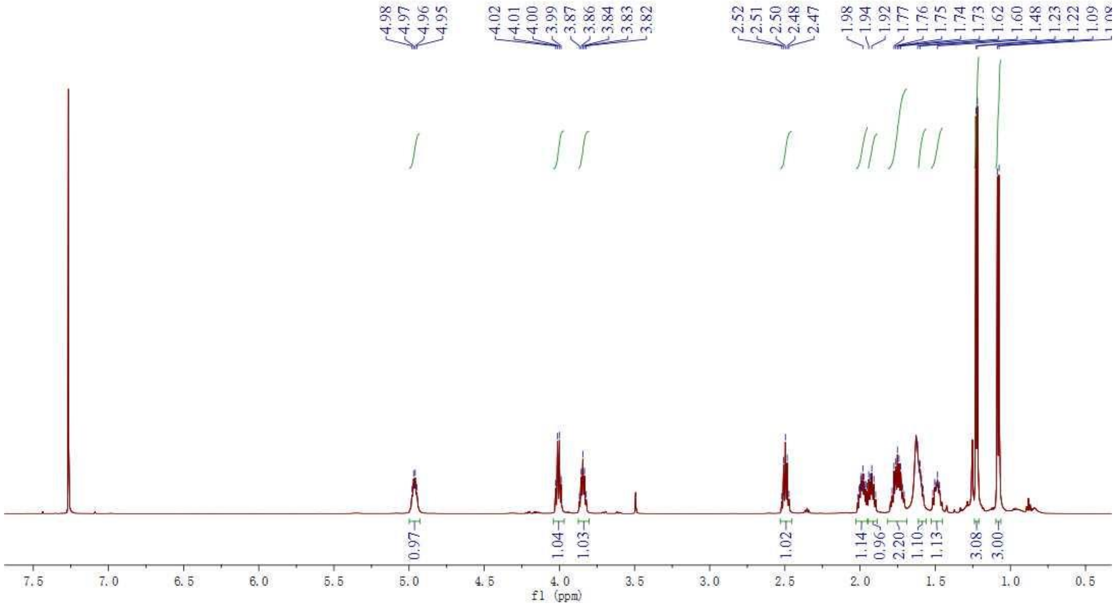


**Figure S1.** $^{1}$H NMR (600 MHz) spectrum of compound **4** in $CDCl_3$

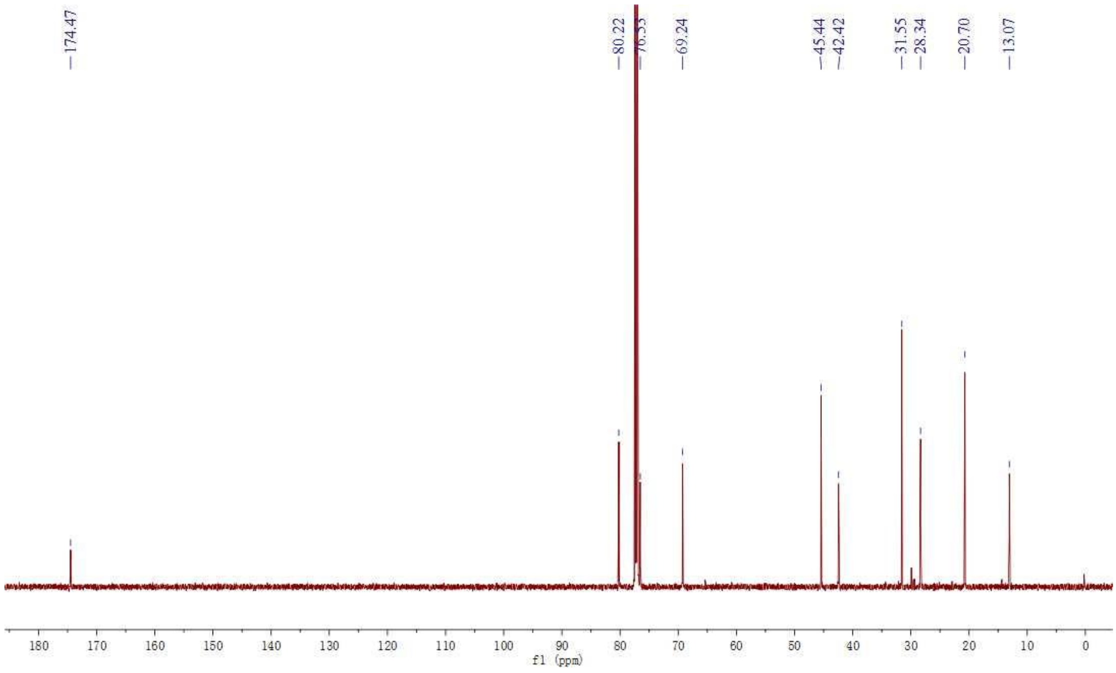


**Figure S1.** $^{13}$C NMR (600 MHz) spectrum of compound **4** in $CDCl_3$

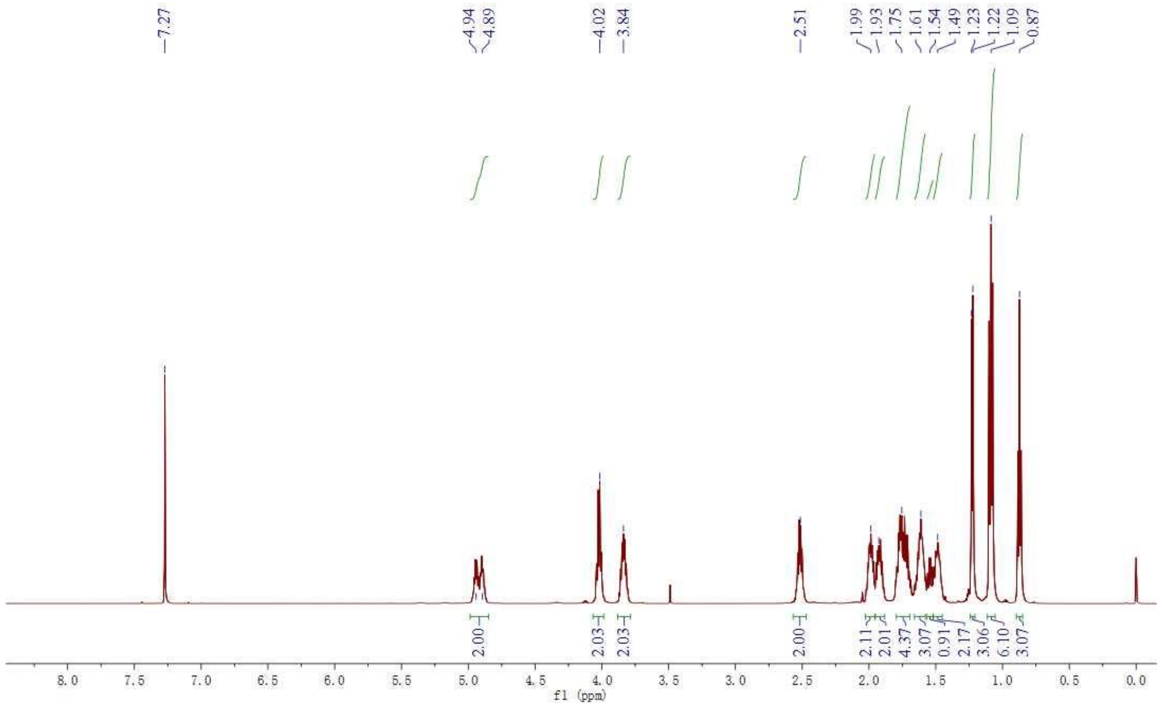

**Figure S1.** $^{1}$H NMR (600 MHz) spectrum of compound **5** in $CDCl_3$

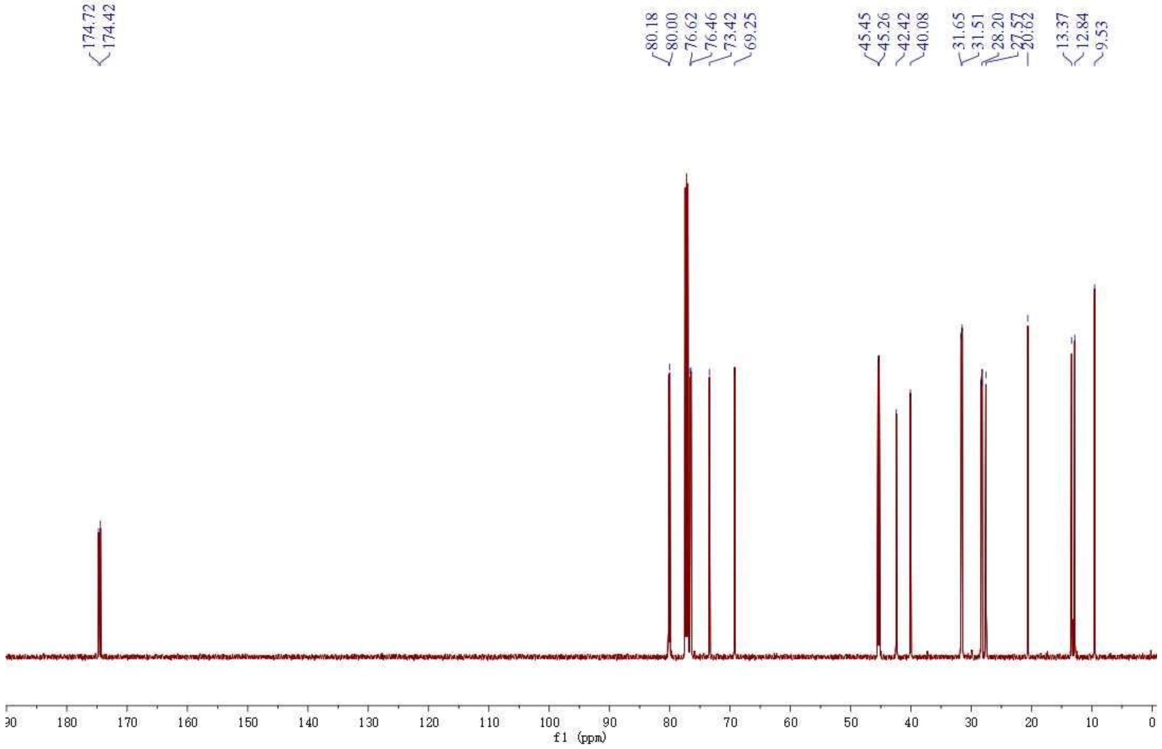

**Figure S1.** $^{13}$C NMR (600 MHz) spectrum of compound **5** in $CDCl_3$

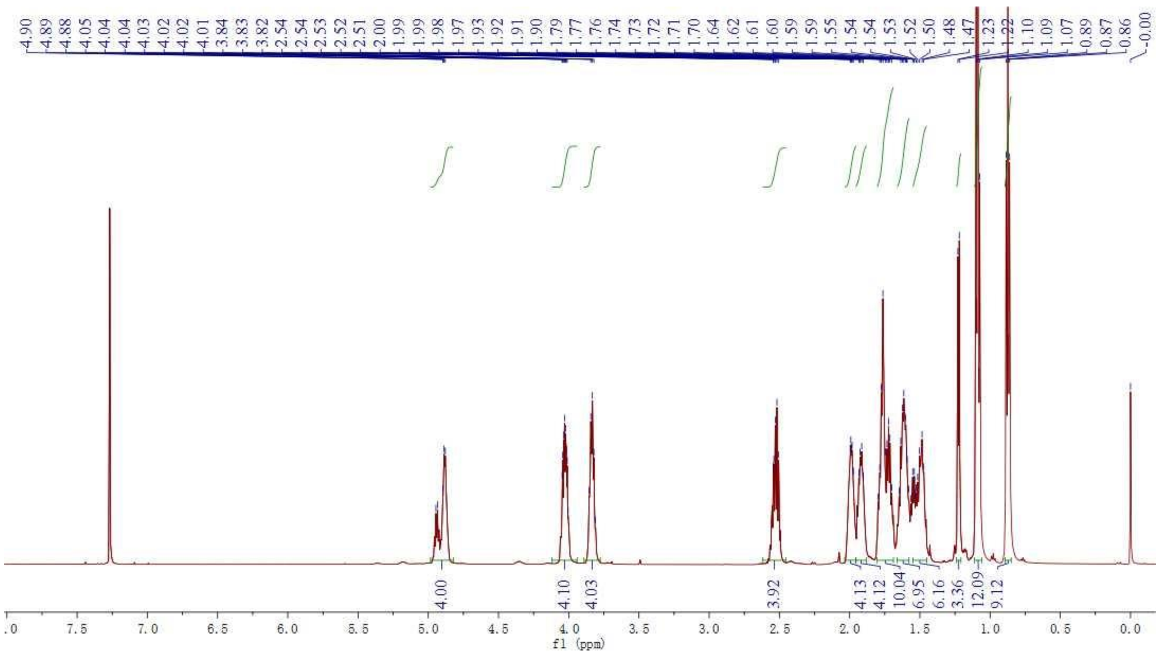

**Figure S1.** $^{1}H$ NMR (600 MHz) spectrum of compound **6** in $CDCl_3$

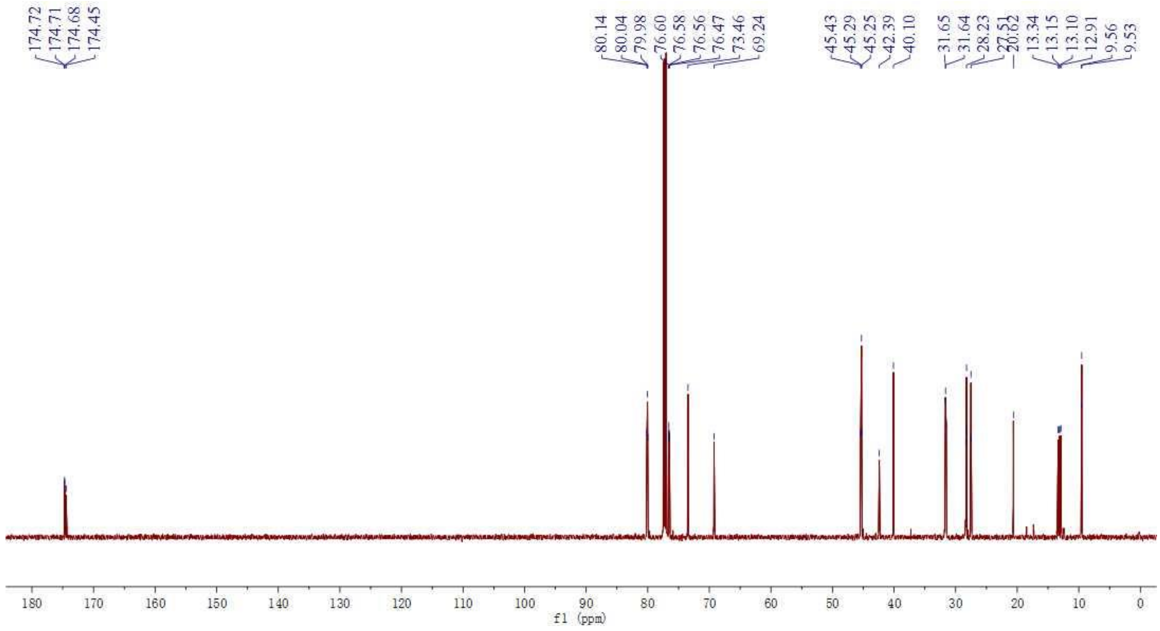

**Figure S1.** $^{13}C$ NMR (600 MHz) spectrum of compound **6** in CDCl3

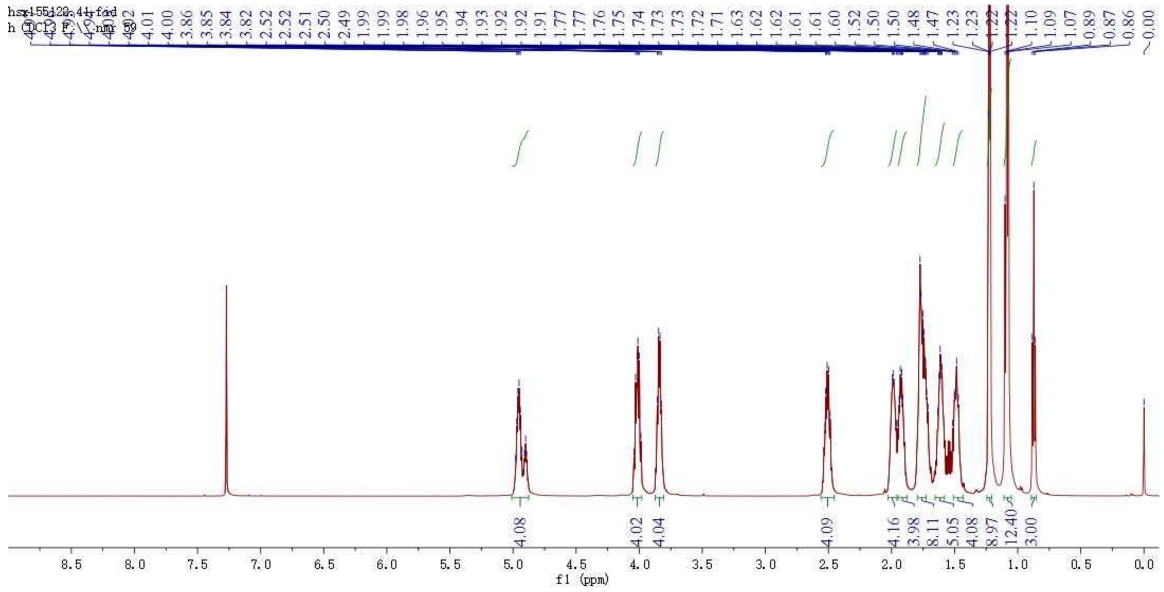

**Figure S1.** $^{13}$C NMR (600 MHz) spectrum of compound **7** in $CDCl_3$

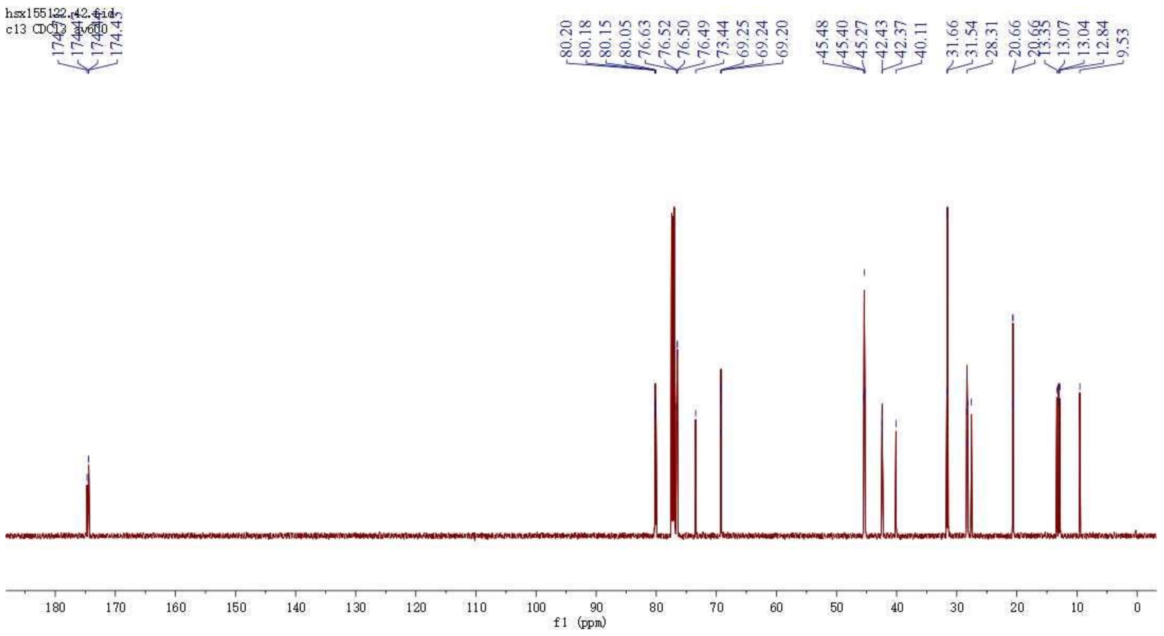

**Figure S1.** $^{13}$C NMR (600 MHz) spectrum of compound **7** in $CDCl_3$

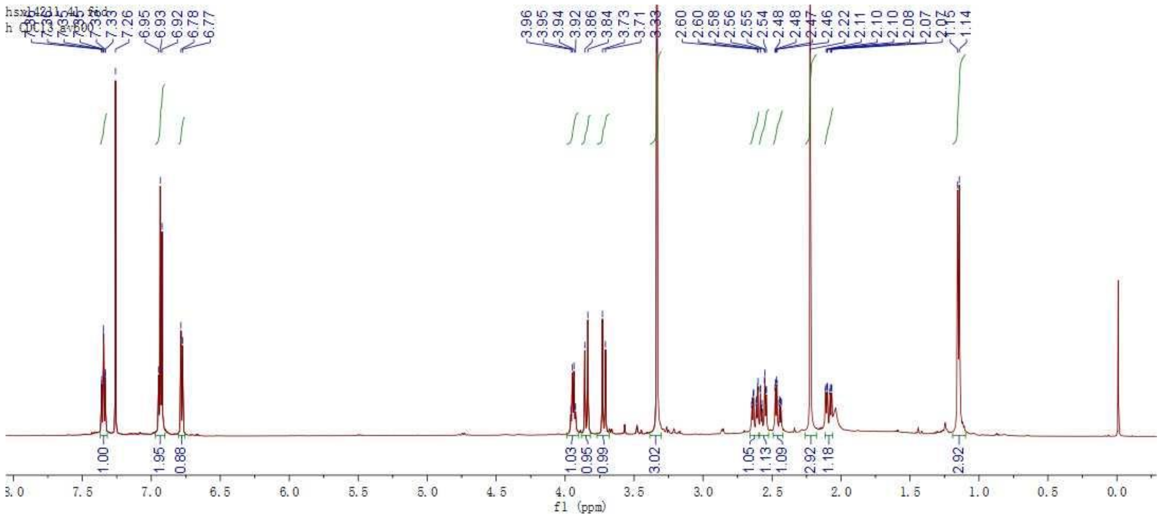


**Figure S1.** $^{1}H$ NMR (600 MHz) spectrum of compound **8** in $CDCl_3$

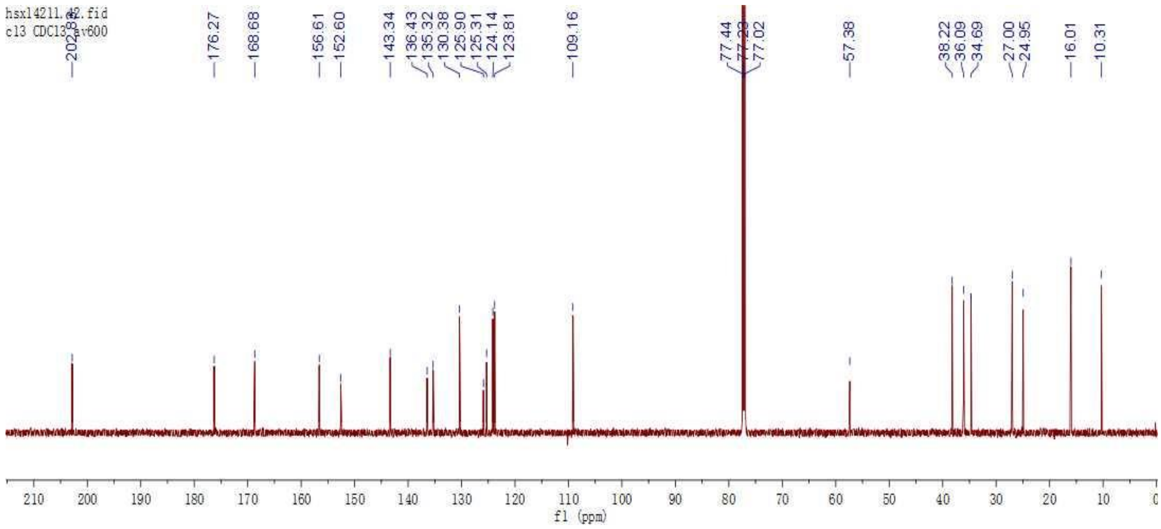


**Figure S1.** $^{13}C$ NMR (600 MHz) spectrum of compound **8** in $CDCl_3$

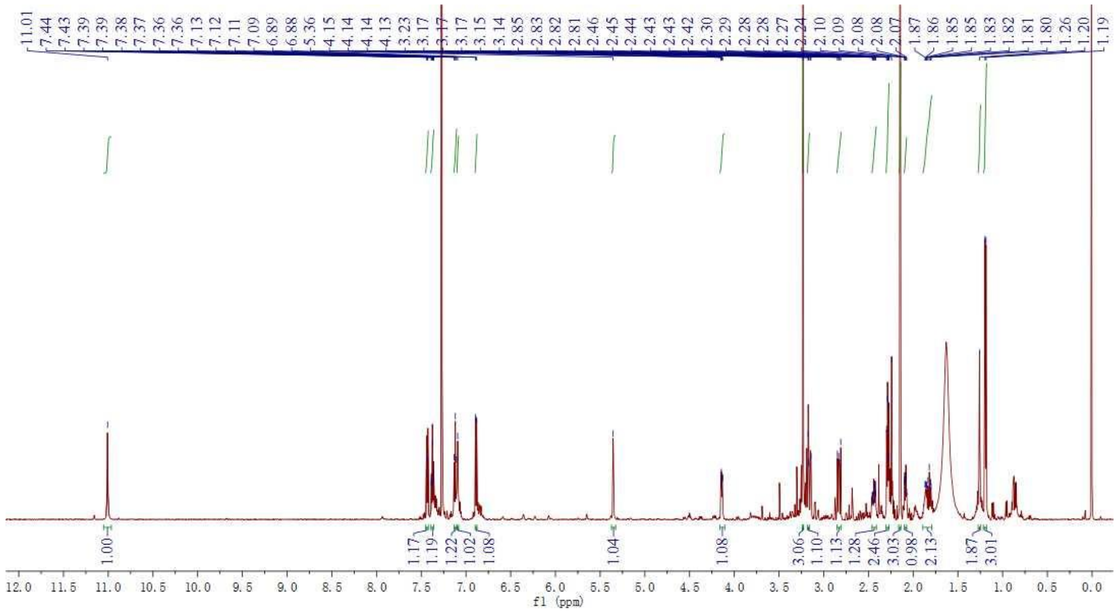

**Figure S2.** $^{1}$H NMR (600 MHz) spectrum of compound **9** in $CDCl_3$

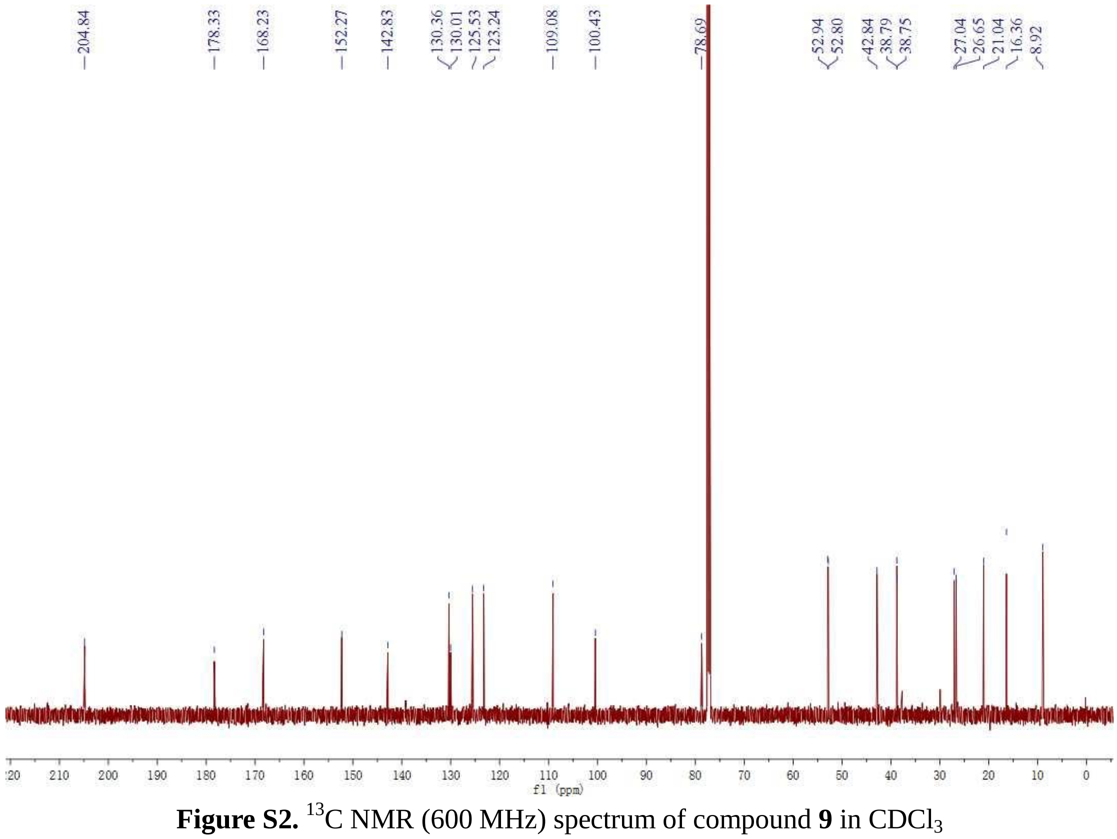

**Figure S2.** $^{13}$C NMR (600 MHz) spectrum of compound **9** in $CDCl_3$

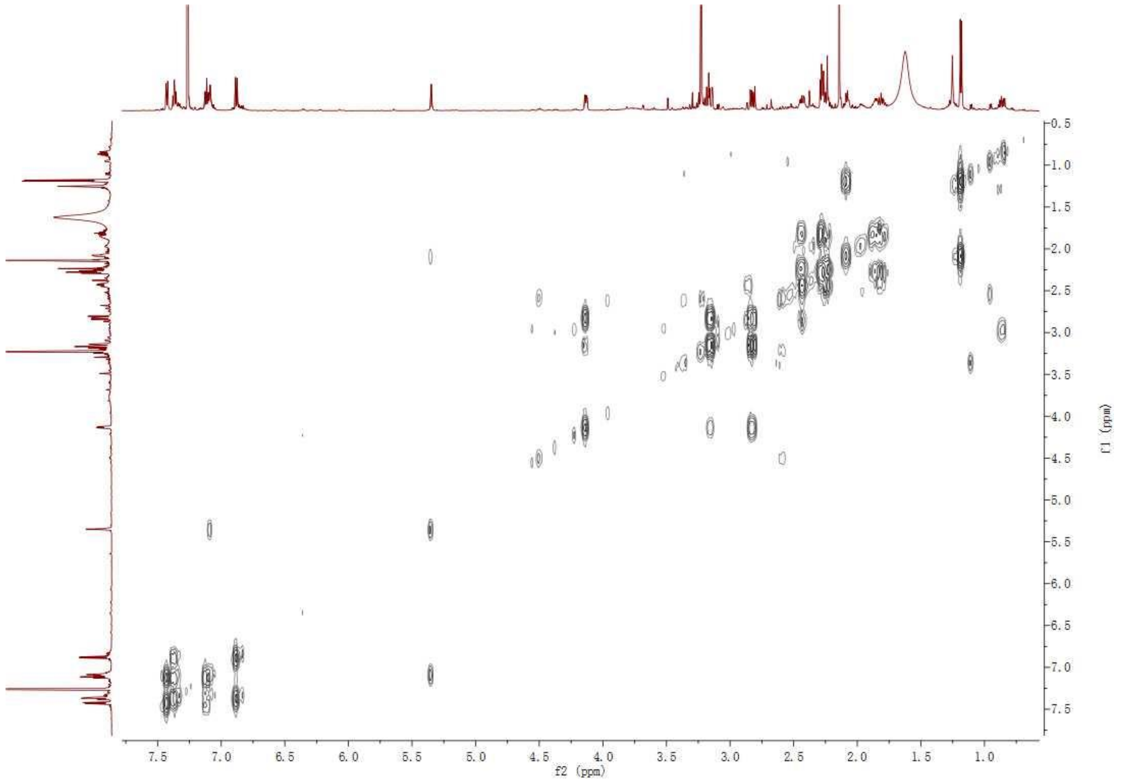


**Figure S2.** $^{1}H$-$^{1}H$ COSY (600 MHz) spectrum of compound **9** in $CDCl_3$

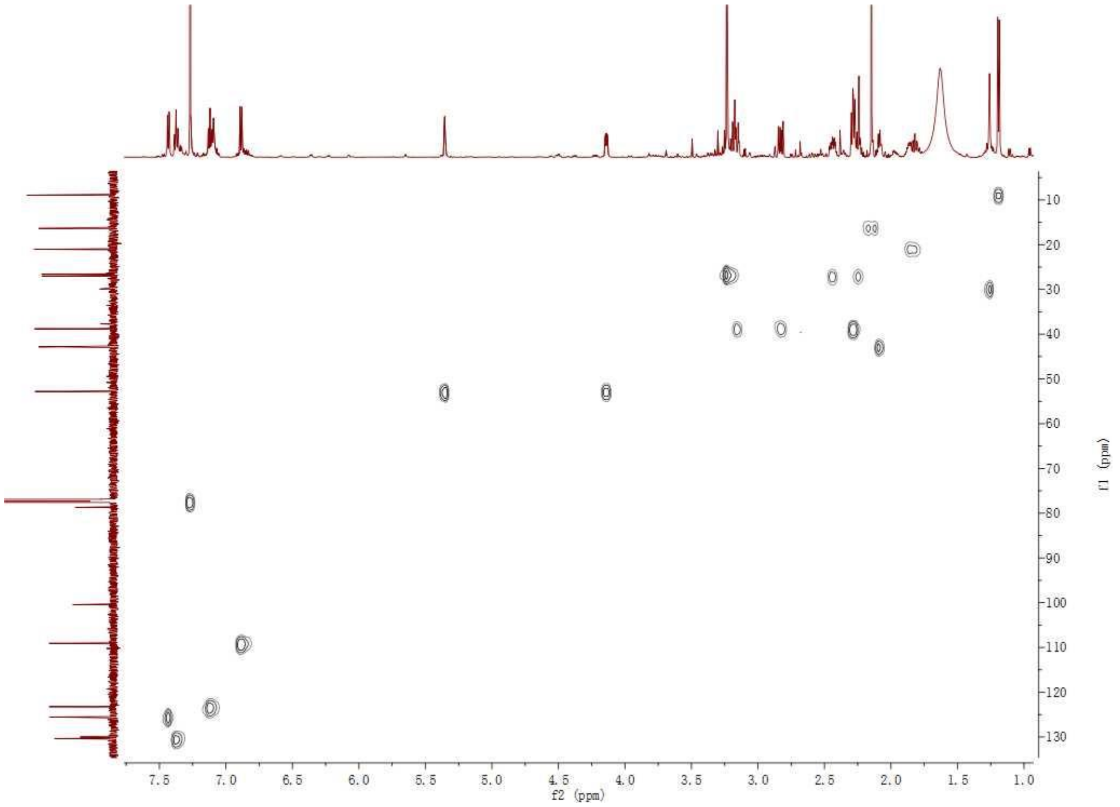


**Figure S2.** HSQC (600 MHz) spectrum of compound **9** in $CDCl_3$

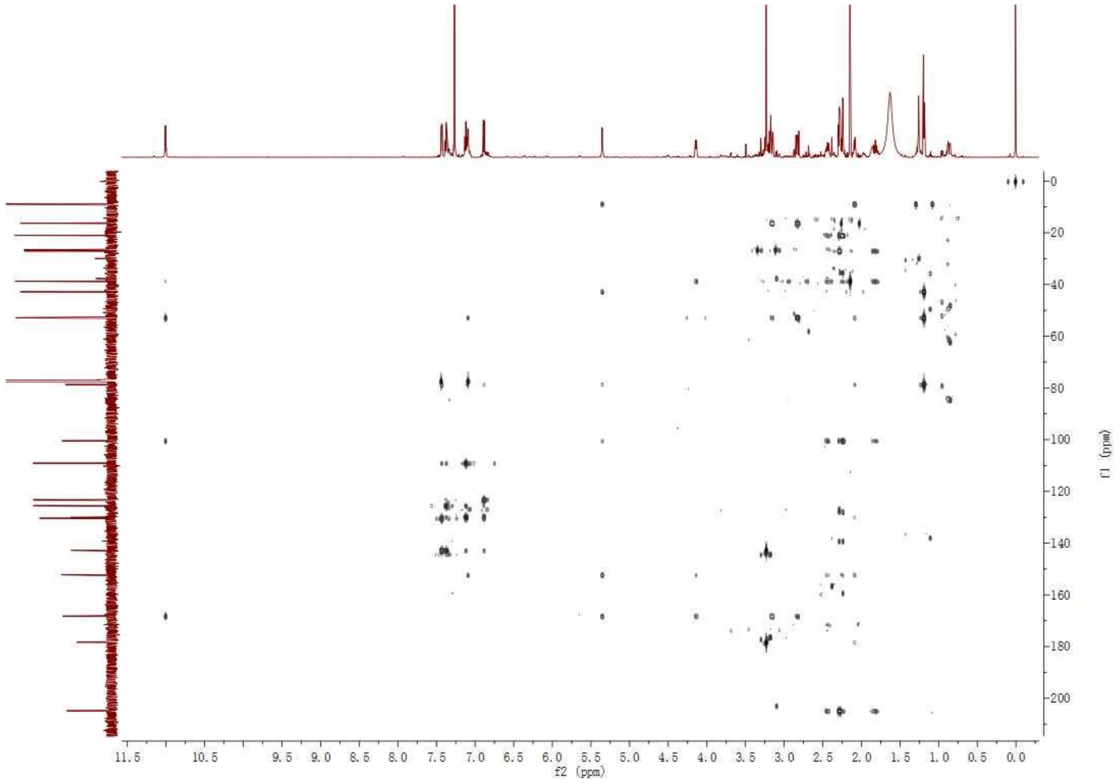


**Figure S2.** HMBC (600 MHz) spectrum of compound **9** in $CDCl_3$

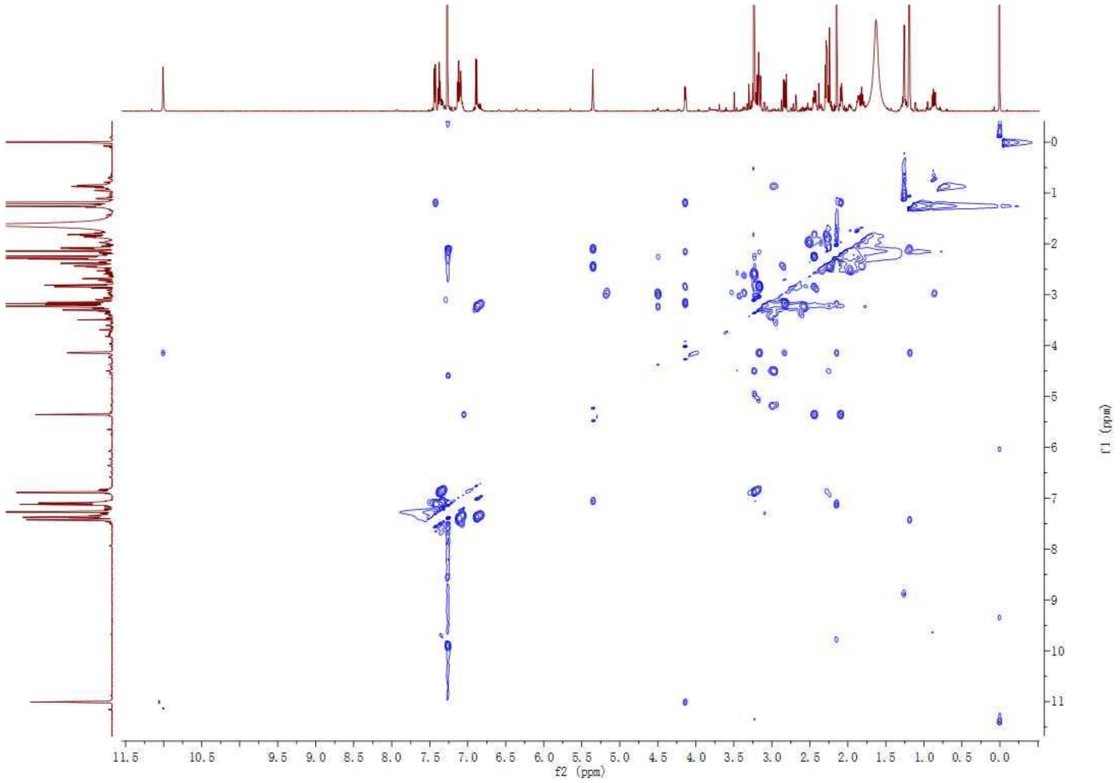


**Figure S2. Roesy** (600 MHz) spectrum of compound **9** in $CDCl_3$

Optical rotation measurement

Model : P-1020 (A060460638)

| No. | Sample | Mode | Data | Monitor<br>Blank | Temp.<br>Cell<br>Temp Point | Date<br>Comment<br>Sample Name | Light<br>Filter<br>Operator | Cycle Time<br>Integ Time |
|---|---|---|---|---|---|---|---|---|
| No.1 | 5 (1/3) | Sp.Rot | -21.5380 | -0.0028<br>0.0000 | 22.5<br>10.00<br>Cell | Wed Sep 12 09:59:35 2018<br>0.00130g/mL MeOH<br>HSA14213 | Na<br>589nm | 2 sec<br>2 sec |
| No.2 | 5 (2/3) | Sp.Rot | -18.4620 | -0.0024<br>0.0000 | 22.5<br>10.00<br>Cell | Wed Sep 12 09:59:40 2018<br>0.00130g/mL MeOH<br>HSA14213 | Na<br>589nm | 2 sec<br>2 sec |
| No.3 | 5 (3/3) | Sp.Rot | -17.6920 | -0.0023<br>0.0000 | 22.5<br>10.00<br>Cell | Wed Sep 12 09:59:46 2018<br>0.00130g/mL MeOH<br>HSA14213 | Na<br>589nm | 2 sec<br>2 sec |

**Figure S2.** Optical rotation data of compound **9** in MeOH

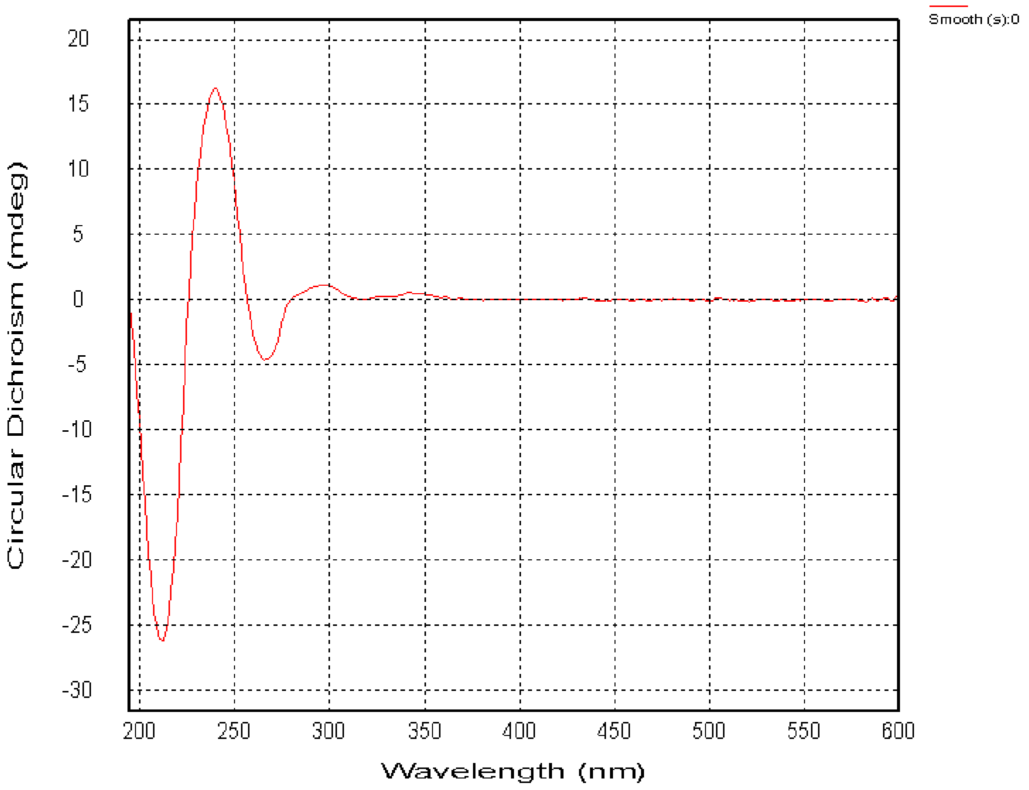


**Figure S2.** CD spectrum of compound **9** in MeOH

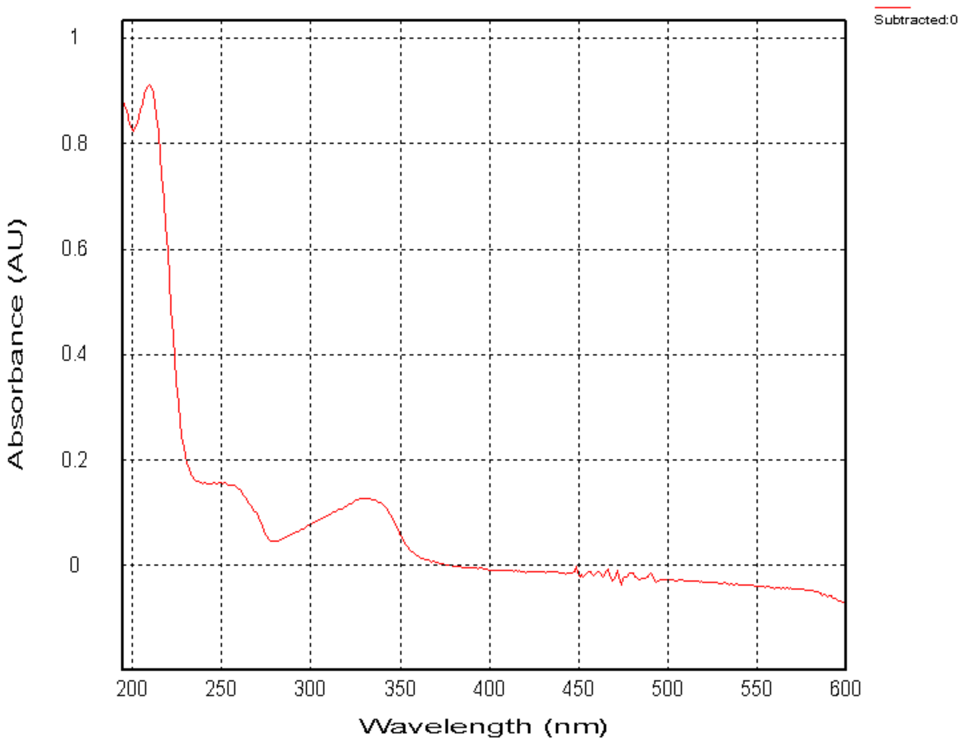


**Figure S2.** UV spectrum of compound **9** in MeOH

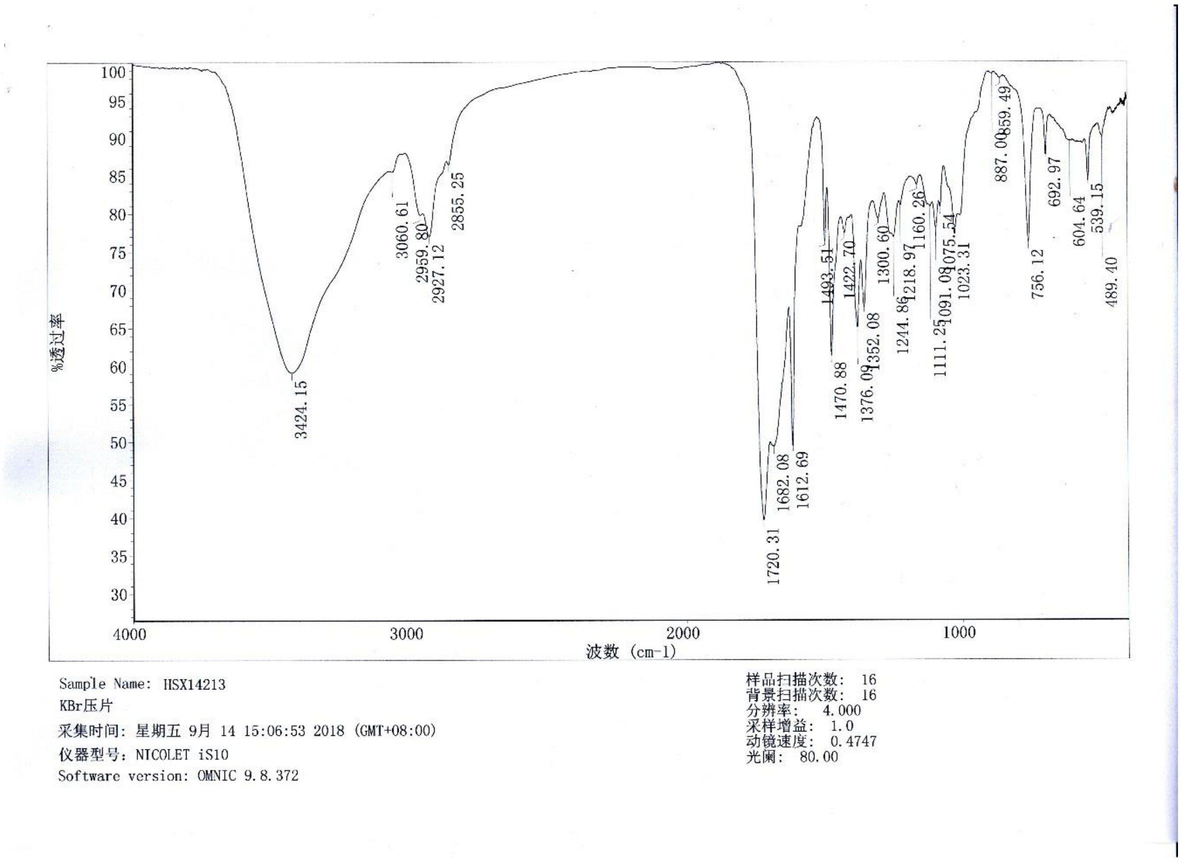


**Figure S2.** IR spectrum of compound **9**

# Qualitative Analysis Report

| | | | |
|---|---|---|---|
| **Data Filename** | HSX14213.d | **Sample Name** | HSX14213 |
| **Sample Type** | Sample | **Position** | P1-A2 |
| **Instrument Name** | Instrument 1 | **User Name** | |
| **Acq Method** | s.m | **Acquired Time** | 7/25/2018 9:41:16 AM |
| **IRM Calibration Status** | Success | **DA Method** | Default.m |
| **Comment** | | | |

**Sample Group** **Info.**
**Acquisition SW Version** 6200 series TOF/6500 series Q-TOF B.05.01 (B5125.2)

## User Spectra

| Fragmentor Voltage | Collision Energy | Ionization Mode |
|---|---|---|
| 100 | 0 | ESI |

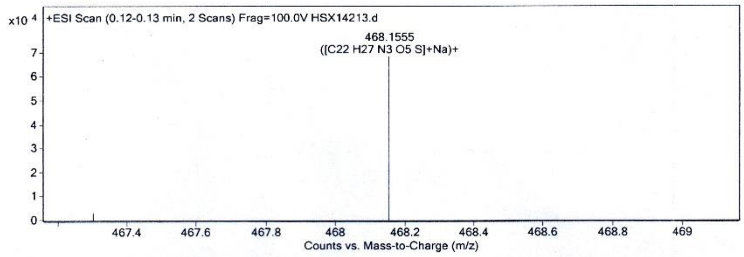


**Peak List**

| *m/z* | z | Abund | Formula | Ion |
|---|---|---|---|---|
| 64.0159 | | 77316.7 | | |
| 274.2745 | 1 | 120462.95 | | |
| 318.3008 | 1 | 135382.3 | | |
| 362.327 | 1 | 63291.14 | | |
| 453.1672 | 1 | 70563.11 | | |
| 468.1555 | 1 | 68312.99 | C22 H27 N3 O5 S | (M+Na)+ |
| 484.1501 | 1 | 117351.11 | | |
| 500.1256 | 1 | 50905.09 | | |
| 913.3232 | 1 | 45645.14 | | |
| 922.0098 | 1 | 46406.51 | | |

**Formula Calculator Element Limits**

| Element | Min | Max |
|---|---|---|
| C | 3 | 60 |
| H | 0 | 120 |
| O | 0 | 30 |
| N | 0 | 5 |
| S | 0 | 1 |

**Formula Calculator Results**

| Formula | CalculatedMass | CalculatedMz | Mz | Diff. (mDa) | Diff. (ppm) | DBE |
|---|---|---|---|---|---|---|
| C22 H27 N3 O5 S | 445.1671 | 468.1564 | 468.1555 | 0.90 | 1.92 | 11.0000 |

--- End Of Report ---



**Figure S2.** HRESI spectrum of compound **9**
